# Gene Biomarkers Related to Th17 Cells in Macular Edema of Diabetic Retinopathy: Cutting-Edge Comprehensive Bioinformatics Analysis and *In Vivo* Validation

**DOI:** 10.3389/fimmu.2022.858972

**Published:** 2022-05-16

**Authors:** Jing Huang, Qiong Zhou

**Affiliations:** Department of Ophthalmology, The First Affiliated Hospital of Nanchang University, Jiangxi Center of National Ocular Disease Clinical Research Center, Nanchang, China

**Keywords:** diabetic retinopathy, diabetic macular edema, Th17 cell, bioinformatic analysis, biomarker

## Abstract

**Background:**

Previous studies have shown that T-helper 17 (Th17) cell-related cytokines are significantly increased in the vitreous of proliferative diabetic retinopathy (PDR), suggesting that Th17 cells play an important role in the inflammatory response of diabetic retinopathy (DR), but its cell infiltration and gene correlation in the retina of DR, especially in diabetic macular edema (DME), have not been studied.

**Methods:**

The dataset GSE160306 was downloaded from the Gene Expression Omnibus (GEO) database, which contains 9 NPDR samples and 10 DME samples. ImmuCellAI algorithm was used to estimate the abundance of Th17 cells in 24 kinds of infiltrating immune cells. The differentially expressed Th17 related genes (DETh17RGs) between NPDR and DME were documented by difference analysis and correlation analysis. Through aggregate analyses such as gene ontology (GO) and Kyoto Encyclopedia of Gene and Genome (KEGG) pathway enrichment analysis, a protein-protein interaction (PPI) network was constructed to analyze the potential function of DETh17RGs. CytoHubba plug-in algorithm, Lasso regression analysis and support vector machine recursive feature elimination (SVM-RFE) were implemented to comprehensively identify Hub DETh17RGs. The expression archetypes of Hub DETh17RGs were further verified in several other independent datasets related to DR. The Th17RG score was defined as the genetic characterization of six Hub DETh17RGs using the GSVA sample score method, which was used to distinguish early and advanced diabetic nephropathy (DN) as well as normal and diabetic nephropathy. Finally, real-time quantitative PCR (qPCR) was implemented to verify the transcription levels of Hub DETh17RGs in the STZ-induced DR model mice (C57BL/6J).

**Results:**

238 DETh17RGs were identified, of which 212 genes were positively correlated while only 26 genes were negatively correlated. Six genes (CD44, CDC42, TIMP1, BMP7, RHOC, FLT1) were identified as Hub DETh17RGs. Because DR and DN have a strong correlation in clinical practice, the verification of multiple independent datasets related to DR and DN proved that Hub DETh17RGs can not only distinguish PDR patients from normal people, but also distinguish DN patients from normal people. It can also identify the initial and advanced stages of the two diseases (NPDR vs DME, Early DN vs Advanced DN). Except for CDC42 and TIMP1, the qPCR transcription levels and trends of other Hub DETh17RGs in STZ-induced DR model mice were consistent with the human transcriptome level in this study.

**Conclusion:**

This study will improve our understanding of Th17 cell-related molecular mechanisms in the progression of DME. At the same time, it also provides an updated basis for the molecular mechanism of Th17 cell crosstalk in the eye and kidney in diabetes.

## Introduction

It is estimated that 34.2% of diabetics worldwide suffer from varying degrees of diabetic retinopathy (DR) (1). Patients with vision-threatening diabetic retinopathy (VTDR) and diabetic macular edema (DME) account for 10.2% and 7.5% of the total diabetic population (2, 3). 3.6% of patients with type 1 diabetes and 1.6% of patients with type 2 diabetes will eventually be blinded by DR and its complications (4). The prevalence is still increasing in some developing countries with large populations (5, 6). There was a significant regional difference in the prevalence of DME between patients with type 1 diabetes mellitus (T1D) and type 2 diabetes mellitus (T2D). The epidemic rate is 11% in Europe, 6% in Southeast Asia and 22% in some African countries (7). The duration of diabetes affects the incidence of DME, with the prevalence of DME ranging from 3% in 10 years to 20% in 20 years or more (7). More than 21 million people around the world were affected by DME (8). Therefore, the discovery of new biomarkers related to the occurrence and progression of DME may provide new and better prospects for the clinical treatment of DME patients.

With the deepening of DR research, autoimmunity was discovered to play a pivotal role in the development of DR (9, 10). Primordial CD4+T cells can be divided into seven subtypes: Th1, Th2, Th17, Treg cells (nTreg and iTreg [Th3 and Tr1]), Th9, Th22, Tfh according to the different types of cytokines secreted in different environments under the stimulation of antigens (11). As a new type of effector CD4+T cell subsets different from Th1 and Th2 cells, the helper T cell 17 (Th17) has become a hot spot in the regulation of research on inflammatory responses in recent years (12). At present, it is believed that RORγt is the main transcriptional regulator of Th17 cells, which can drive the expression of IL-17A, IL-17F and IL-22. These three cytokines are the three landmark cytokines secreted by Th17 cells. Other transcription factors involved in Th17 differentiation, such as RORα, Batf and IRF4, may also be involved in regulating the expression of IL-17 gene (13). Th17 cells can not only enhance immune defense and maintain immune homeostasis in a non-inflammatory way, but also have “pathogenic” phenotype and widely participate in the occurrence and development of a variety of inflammatory diseases (14). At present, the research on Th17 cells in DR is still rare, and the research on its mechanism of DME generation and progression in DR has not been reported. Therefore, in-depth exploration of the molecular regulation mechanism of Th17 cell pathogenicity is helpful to provide new ideas for the treatment of DR and DME.

With the emergence and popularization of gene chip and RNA-seq technology, the search for new diagnostic markers and therapeutic targets for DR and DME becomes more concise. Some genetic biomarkers related to DR and DME have been reported with the help of these techniques (15–17). However, the relationship between Th17 cells and their related biomarkers and the molecular mechanism of Th17 cells involved in DR and DME have not been explored. In order to study the relationship between them, the ImmucellAI algorithm was used to calculate the composition of 24 kinds of immune cells, including Th17 cells. The R software was used to calculate the differential genes and correlation analysis. After comprehensive calculation, the differentially expressed Th17 cell related genes (DETh17RGs) in non-proliferative diabetic retinopathy (NPDR) and diabetic macular edema (DME) were obtained. Then enrichment analyses of DETh17RGs were implemented to obtain functions and pathways annotation, protein-protein interaction network (PPI) was constructed, and Hub DETh17RGs were obtained by Cytoscape software analysis. Subsequently, multiple DR-related independent datasets were obtained in the datasets for verification, and GSVA scoring method was used to integrate all hub genes to verify the diagnostic efficacy of diabetic nephropathy (DN)-related independent datasets. Finally, Hub DETh17RGs were applied to the retina of molded animals for verification. Through the above analyses and experiments, we can obtain the genetic molecular mechanism of the occurrence and development of DME related to Th17 cells, and explore the crosstalk association related to the occurrence and development of diabetes in DME and DN, so as to provide new ideas for the study of the mechanism of Th17 cells in diabetes.

## Materials and Methods

### GEO Dataset Processing

The GSE160306 dataset, which is based on the GPL20301 Illumina HiSeq 4000 (Homo sapiens) platform, was downloaded from the Gene Expression Omnibus (GEO) database (https://www.ncbi.nlm.nih.gov/geo/). The dataset is high-throughput sequencing expression profile analysis data, including 79 transcriptome data related to diabetic retinopathy. The gene expression profile data of macular samples of different clinical stages: NPDR (9 samples) and DME (10 samples) were analyzed. Before the analysis, Log2 was used to normalize the high-throughput data.

### Immunocyte Infiltration in Macular Samples of NPDR and DME

The Immune Cell Abundance Identifier (ImmuCellAI) was used to estimate the abundance of 24 variety types of infiltrating immune cells including Th17 cells from RNA-seq digital gene expression matrix data and to obtain the corresponding immune cell infiltration matrix (18). Landscapes of immune cell infiltration in retina tissue from macula of NPDR patients and retina tissue from macula of DME patients were downloaded. The proportion of each immune cell subtype was extracted from the sample. A heat map that included the 24 types of immune cells was created using the pheatmap package. The total infiltration score of each sample is defined as 1, which is the sum of the percentage of 24 infiltrating immune cells. In order to compare the abundance of each immune cell type between NPDR and DME samples, the Wilcoxon rank sum test was applied.

### Identification of DETh17RGs

The “edgeR” package in R software was used to identify and obtain differentially expressed genes (DEGs) between NPDR and DME samples. The criteria for statistical significance were: |logFC| (fold change) > 1.0 or p < 0.05. Then, Pearson correlation analysis was implemented to determine the genes related to Th17 cell abundance. The Pearson’s correlation coefficient (PCC) of DEGs >0.6 were regarded as DETh17RGs.

### Functional Enrichment Analysis

ShinyGO (http://bioinformatics.sdstate.edu/go/) is an integrated program that can be accessed through the Internet to display the results of genetic enrichment analysis and the visualization of gene characteristics. Its purpose is to explore the potential biological denotation of gene clusters for users. Therefore, in order to investigate the biological function and denotation of DETh17RGs, the biological process of gene ontology (GO) and the Kyoto Encyclopedia of Gene and Genome (KEGG) pathway were analyzed and enriched by utilizing ShinyGO online software. The enrichment analysis significance threshold was set as the false discovery rate (FDR) < 0.05.

### Screening of Hub DETh17RGs by Comprehensive Method

In this study, three methods were used to screen Hub DETh17RGs: CytoHubba plug-in algorithm in Cytoscape software, lasso regression analysis and support vector machine recursive feature elimination (SVM-RFE). The CytoHubba plug-in has the ability to rank and filter nodes in the network according to the characteristics of network in order to identify the core elements of the complex network. All eleven algorithms including MCC, DMNC, MNC, Degree, EPC, EcCentricity, Closeness, Radiality, Betweenness, Stress, and BottleNeck in the CytoHubba plug-in were applied and the intersection of the top 50 nodes from each method were recorded to identify the latent Hub DETh17RGs. In the CytoHubba plug-in, the DETh17RGs with eleven algorithm conditions that meet nine or more conditions were screened and considered as candidate Hub DETh17RGs. LASSO regression was used to minimize extra redundancy and irrelevance in order to achieve the purpose of sparse and feature selection. R software package glmnet was used in lasso-cox method for regression analysis, and the response type was set to binomial. In addition, 3-fold cross-validation was set to adjust the penalty parameters, and variables were determined by finding lambda (λ) with the minimum classification error. SVM-RFE is an optimal feature selection algorithm, which is based on support vector machine and sorts features according to recursive feature deletion sequence. The SVM-RFE classifier made by R package e1071 was used to classify and analyze the candidate biomarkers. The SVM-RFE model based on radial basis function and 10-fold cross-validation was established, and the best variables were selected according to the minimum 10 × CV error. Finally, the candidate genes obtained by the above three methods were intersected to attain the final Hub DETh17RGs.

### PPI Network Construction

The development of protein-protein interaction (PPI) network was demonstrated with STRING (search tool for searching interacting genes/proteins), which can provide systematic screening of interactions between human proteins and genes. The DETh17RGs list obtained by difference and correlation analysis was uploaded to the STRING database to identify and integrate to build a PPI network with a default comprehensive score of more than 0.4. Export the PPI network file and revisualize it using the Cytoscape 3.9.0 software.

### Verification of Hub DETh17RGs in Proliferative Diabetic Retinopathy

To study the relationship between Hub DETh17RGs and the progress of PDR, the unsupervised hierarchical clustering analysis of the GSE160306 dataset was implemented by performing the “Pheatmap” package in R. Three independent datasets containing samples extracted from the PDR neovascularization membrane were used to verify the expressions of the Hub DETh17RGs in the extramacular proliferative membrane, that is, GSE94019 (n=13), GSE102485 (n = 5), and GSE60436 (n=6). In order to establish the comparison standard of the verification set, the control group in the dataset GSE160306 was selected as the normal person without diabetes, and the DME group remained unchanged, and the split violin plot was performed to visualize the comparison between the two groups.

### GSVA Constructs Hub DETh17RGs Diagnostic Features

Gene set variation analysis (GSVA) is a nonparametric and unsupervised method, which combines the gene expression profile of RNA-seq and the gene set of metabolic pathway to evaluate the metabolic pathway enrichment score of each sample. The matrix of gene X samples were transformed into the matrix of gene set X samples, and the enrichment score was calculated. In this study, the Hub DETh17RGs were integrated as the set of characteristic genes to participate in each validation dataset, and the R package GSVA was used to calculate the GSVA enrichment score for each sample, which we define as the “Th17RG score”. Because of the high clinical correlation between DR and DN (19, 20), DN has been proved to be an independent risk factor for DR and DME (20–22). In this study, the validation set includes not only the above three PDR sample datasets from intraocular neovascularization membrane, but also three tissue sample datasets from the kidneys of patients with diabetic nephropathy (DN). The datasets were GSE142025 (n=27), GSE30528 (n=22) and GSE96804 (n=61). The package pROC in R was performed to analyze and assess the diagnostic value of the Hub DETh17RGs in GSE30528 and GSE96804 using receiver operating characteristic (ROC) curves and reckoning the area under the curve (AUC). To investigate the relationship between Hub DETh17RGs and DN disease progression, the unsupervised hierarchical clustering analysis was performed again on the GSE142025 dataset using the R package “Pheatmap”.

### Isolation of RNA and Mensuration of Hub DETh17RGs Expression by Quantitative Real-Time Polymerase Chain Reaction

Eight-week-old C57BL/6J mice (22.5-26g) were fed standard pellet diet without restriction on diet and water. The indoor atmospheric conditions was good, the temperature was controlled at (23 ± 2) °C, and the relative humidity was 50%. Blood glucose was detected before modeling (normal blood glucose was between 4.25 and 6.50 mmol/L). STZ solution was prepared by dissolving streptozotocin (STZ, Sigma Company, USA, S0130-1G) in sodium citrate solution of 0.1 mol/L at pH 4.2. Mice were intraperitoneally injected with 55mg/kg STZ solution for 5 consecutive days, fasted for 6 hours before injection, and blood glucose was measured by tail vein 7 days after the last injection. If blood glucose was > 16.5mmol/L, the diabetic mouse model was considered to be established successfully. Five months after the successful construction of the diabetic mouse model, hyperplasia and disorder of retinal capillaries were observed, and proliferative blood vessel groups were observed in the ganglion cell layer and the inner core layer, indicating that typical characteristic pathological changes of DR had appeared, and the DR mouse model was recognized to be successfully constructed.

In addition to obtaining Hub DETh17RGs through comprehensive bioinformatic analyses, animal experiments were also organized to verify the expression level of Hub DETh17RGs in the retina. The mice modeled by the above methods were used as the experimental subjects, in which 8 mice were successfully modeled as the experimental group and 6 mice were classified as the control group by intraperitoneal injection of normal saline. Total retinal tissue RNA was extracted using TRIzol extraction reagent (TRIzol; Invitgen,Carlsad, CA) and reverse transcribed with the HiFiScript cDNA synthesis kit (First-Strand,Cowin Biosciences, China) to detect the expression level of Hub DETh17RGs-mRNA in the mouse retina. The primers in the experiment were synthesized by Shanghai Biotechnology Company (Shanghai, China). The sequence information of the primers was displayed in **Table 1**. According to the scheme recommended by the manufacturer, SYBGREEN PCR Master Mix’s PCR system kit (Conway Century Co., Ltd., Beijing, China) was used for real-time quantitative PCR reaction. The internal reference gene used to measure the level of gene expression in this study is GAPDH. The relative expression level of Hub DETh17RGs was estimated by two power values of Δ Ct, and the experiment of each gene in each sample was repeated 3 times.

**Table 1:** Primers for Hub DETh17RG.

Genes	Primer	Sequences
Cd44	Forward	CGGAACCACAGCCTCCTTTCAA
	Reverse	TGCCATCCGTTCTGAAACCACG
Cdc42	Forward	GATTGGTGGAGAGCCATACACTC
	Reverse	TGAGGATGGAGAGACCACTGAG
Timp1	Forward	TCTTGGTTCCCTGGCGTACTCT
	Reverse	GTGAGTGTCACTCTCCAGTTTGC
Bmp7	Forward	GGAGCGATTTGACAACGAGACC
	Reverse	AGTGGTTGCTGGTGGCTGTGAT
Rhoc	Forward	GAGGCAAGATGAGCATACCAGG
	Reverse	GCCATCTCAAATACCTCCCGCA
Flt1	Forward	TGGATGAGCAGTGTGAACGGCT
	Reverse	GCCAAATGCAGAGGCTTGAACG
Gapdh	Forward	CATCACTGCCACCCAGAAGACTG
	Reverse	ATGCCAGTGAGCTTCCCGTTCAG

## Results

### DETh17RGs in the Formation and Progress of DME

**Figure 1:**
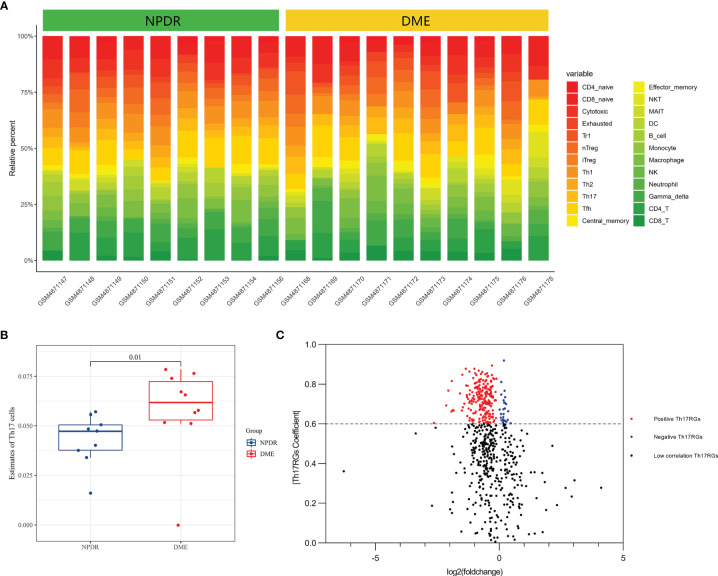
Macular DETh17RGs were identified between the NPDR group and the DME group. **(A)** abundance of 24 types of immune cells in 19 samples calculated using the ImmucellAI algorithm; **(B)** comparison of the total Th17 percentage between 9 NPDR samples and 10 DME samples; **(C)** volcano plot of differentially expressed genes between 9 NPDR samples and 10 DME samples. DETh17RGs, differentially expressed Th17 cell related genes, Th17RGs, Th17 cell related genes.

Using the ImmucellAI algorithm to calculate the immune cell composition of each sample in the GSE160306 dataset, **Figure 1A** showed the component results obtained from 19 DR macular samples. DME group showed significantly higher Th17 cell infiltration than NPDR group (**Figure 1B**). A total of 2216 DEGs were obtained by differential analysis with the edgeR package, of which 238 DEGs were highly correlated with Th17 cell abundance (PCC > 0.6) and were identified as DETh17RGs (**Figure 1C**). Of all the immune cell species analyzed, Th17 cells simultaneously had significant differences in NPDR versus normal and NPDR versus DME, indicating a critical role for Th17 cells in the progression of DR and DME.

### GO and KEGG Enrichment Analysis of DETh17RGs

**Figure 2:**
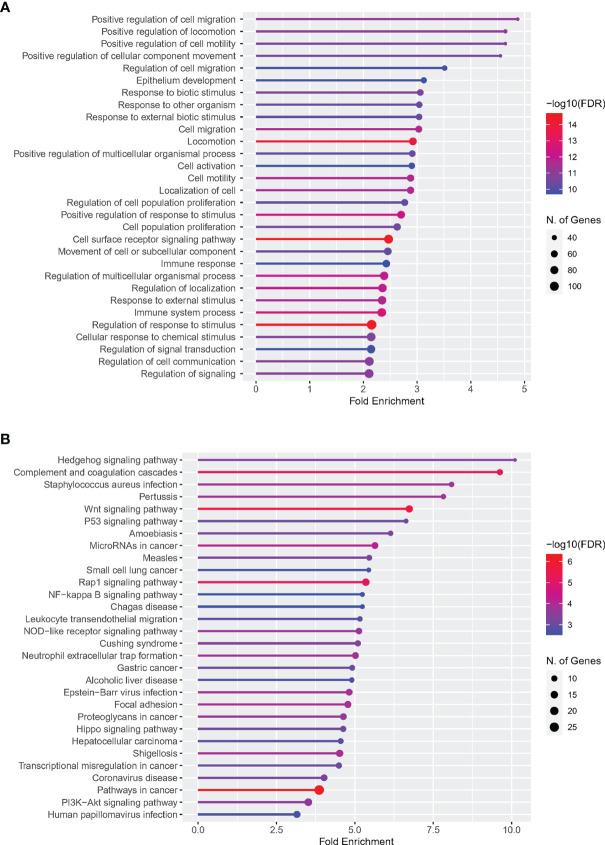
Enrichment analysis of DETh17RGs in GO and KEGG pathways. **(A)** Top 30 terms of DETh17RGs enrichment in the biological process of GO; **(B)** Top 30 terms of DETh17RGs enriched in KEGG pathway. Fold enrichment = GeneRatio/BgRatio.

The web page analysis tool ShinyGO was used to enrich and analyze the uploaded DETh17RGs list. The top five terms ranked by Fold Enrichment in the biological process of the GO analysis were: Positive regulation of cell migration, Positive regulation of locomotion, Positive regulation of cell motility, Positive regulation of cellular component movement and Regulation of cell migration. Due to space limitations, GO terms related to Th17 cells cannot be shown in the figure. We present them in text form as follows: regulation of interleukin 17 production, positive regulation of interleukin 17 production, and T-helper cell type 17 immune response. The same ranking method in the KEGG analysis of the top five terms were: Hedgehog signaling pathway, Complement and coagulation cascades, Staphylococcus aureus infection, Pertussis, Wnt signaling pathway. The details of the first 30 terms of GO and KEGG were visualized in **Figures 2A, B**.

### Identification of Hub DETh17RGs

**Figure 3:**
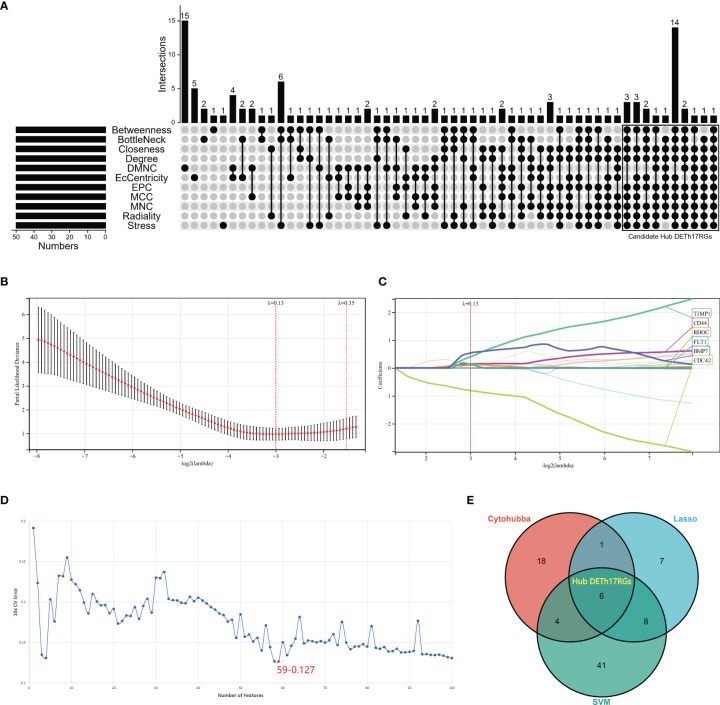
The screening processes of the Hub DETh17RGs. **(A)** the candidate Hub DETh17RGs obtained by using eleven algorithms of the CytoHubba plug-in in Cytoscape software, the genes that meet the conditions of nine or more algorithms were recognized as candidate Hub DETh17RGs and were illustrated in the black box in the figure. **(B)** The parial likelihood deviance in the jackknife rates analysis. **(C)** The Lasso coefficient distribution plot of Hub DETh17RGs was used to identify the eigenvalues of the constructed diagnostic signal. Each curve corresponds to a candidate Hub DETh17RG. **(D)** The SVM-RFE algorithm identifies the latent Hub DETh17RG with the lowest error rate (10 × CV error rate = 59-0.127) and the highest precision. **(E)** Venn diagram showed the Hub DETh17RGs obtained by taking the comprehensive intersection of the three methods: CytoHubba, Lasso regression, and SVM-RFE.

The upsetR method was used to obtain the intersection of the genes obtained by 11 methods in the CytoHubba plug-in, and there were 29 candidate Hub DETh17RGs that met the intersection condition of 9 or more (**Figure 3A**). In Lasso regression analysis, the candidate Hub DETh17RGs for accurate prediction of DME can be determined when λ = 0.13 in the process of constructing LASSO model (**Figure 3B**). Based on the optimal λ value of 0.13, we plotted the LASSO coefficient spectrum of differentially expressed genes (**Figure 3C**), and a total of 22 potential Hub DETh17RGs were obtained. In the analysis of the SVM-RFE algorithm, all DETh17RGs were screened and the top 100 genes were chosen to construct the SVM-RFE model. Check that the error rate of model 10x CV was 0.127 when the number of features was 59, that is, when the red dot position was the lowest error rate, and these 59 genes were included in the candidate Hub DETh17RGs (**Figure 3D**). After the intersection of the genes obtained by the above three methods, there were still 6 genes left, which were defined as Hub DETh17RGs (**Figure 3E**). The Hub DETh17RGs were CD44, CDC42, TIMP1, BMP7, RHOC, FLT1.

### PPI Network and Identification of Hub DETh17RGs

**Figure 4:**
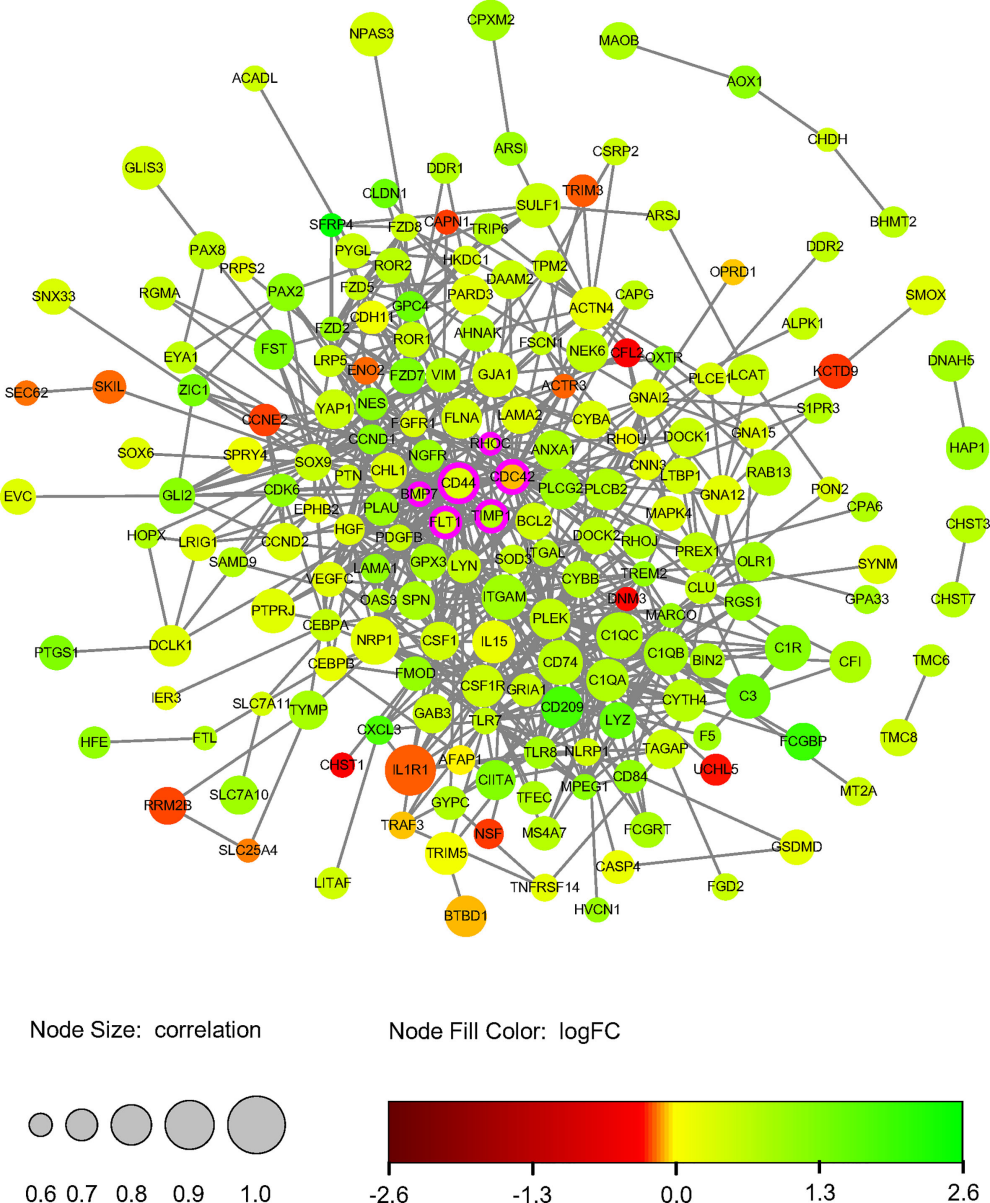
DETh17RGs’ protein-protein interaction network. The circular diameter of the node represents the Pearson correlation coefficient between a specific gene and Th17 cell abundance. Nodes with rosy contours stand for hub genes, green nodes represent positive logFC and red nodes represent negative logFC. FC, fold change.

A DETh17RGs protein-protein interaction network consisting of 195 nodes and 695 edges was created by utilizing STRING online network tool and was re-visualized with Cytoscape (**Figure 4**). After calculation, the degrees of Hub DETh17RGs were CD44 (degree = 43), CDC42 (degree = 35), TIMP1 (degree = 14), BMP7 (degree = 12), RHOC (degree = 15) and FLT1 (degree = 14). Compared with NPDR, there were 5 Hub DETh17RGs up-regulated and 1 down-regulated in DME.

### Verification of Hub DETh17RGs in PDR

**Figure 5:**
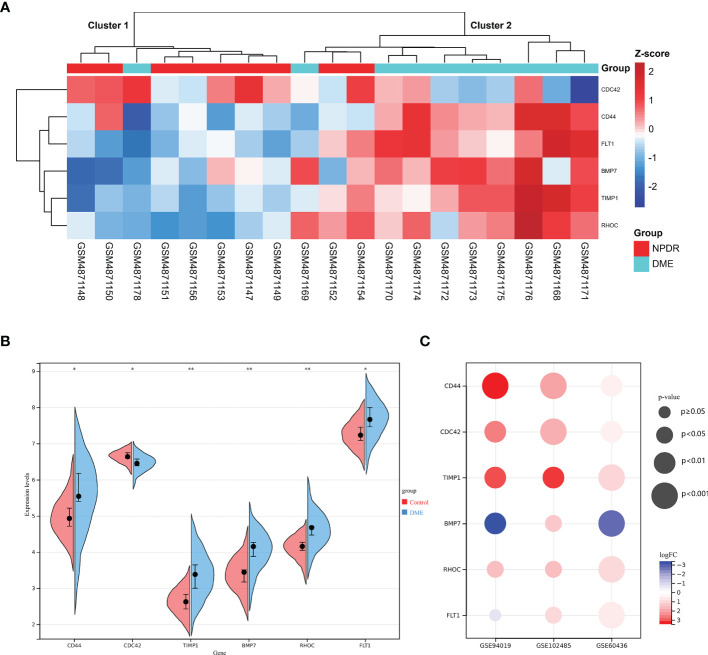
The expression pattern of DETh17RGs in DR. **(A)** the unsupervised hierarchical clustering heat map of the six hub DETh17RGs in the dataset GSE160306, reflecting the difference of z-score between NPDR group and DME group; **(B)** the difference between normal group and DME group of the six hub DETh17RGs in GSE160306. **(C)** the verification of DETh17RGs between normal retina and PDR proliferative membrane in different independent datasets. *p < 0.05, **p < 0.01.

Hub DETh17RGs expression data were extracted from GSE160306 datasets for unsupervised hierarchical clustering. According to the results of ward.D2 clustering method, most of the NPDR samples were divided into cluster 1 (7/9,78%), while the vast majority of DME samples were classified as cluster 2 (8/10,80%), which fully showed that these six genes were closely related to the occurrence and development of DME (**Figure 5A**). Visualization of Hub DETh17RGs expression in the GSE160306 dataset with split violin plot revealed that all the six genes illustrated significant differences between normal and DME (**Figure 5B**). During the verification process of datasets, we found that except for BMP7, the expression patterns of the other five genes in the validation set were basically the same: CD44, CDC42, TIMP1, RHOC were significantly up-regulated in all validation datasets, FLT1 was significantly up-regulated in all validation datasets except GSE94019, and BMP7 was significantly up-regulated in GSE102485, but significantly down-regulated in GSE94019 and GSE60436 (**Figure 5C**).

### Diagnostic Value of Hub DETh17RGs Features in DN

**Figure 6:**
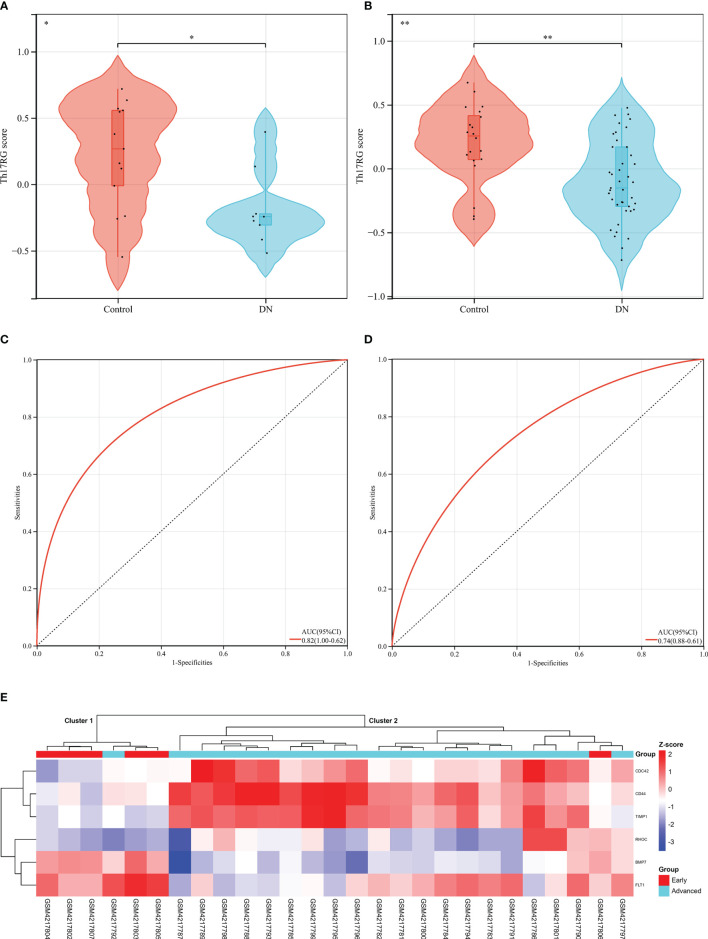
Diagnostic efficacy of the Th17RG score in patients with DN. **(A, B)** Comparison of Th17RG scores between normal and DN patients in the GSE30528 and GSE96804 datasets. **(C, D)** Receiver operation characteristic curve for diagnosing DN plotted with Th17RG score in the GSE30528 and GSE96804 datasets. **(E)** The unsupervised hierarchical clustering heat map of six hub DETh17RGs in the GSE142025 dataset, which shows the z-score difference between the early DN and the advanced DN. AUC, area under the curve. * means *p* < 0.05, ** represents *p* < 0.01

Since diabetes involves multiple systems and multiple organs, Th17 cells can also infiltrate in multiple organs under pathological conditions, and DR and DN are independent risk factors for each other, these reminded us that Hub DETh17RGs may also contribute to the identification of DN patients. In order to verify this assumption, six Hub DETh17RGs were aggregated and the GSVA algorithm was assigned to analyze and calculate the Th17RG score. In GSE30528 and GSE96804, the Th17RG score of DN patients was significantly lower than that of normal controls (**Figures 6A, B**). ROC analysis showed that the AUC of GSE30528 and GSE96804 reached 82% and 74%, respectively, which could be considered to have a high degree of differentiation (**Figures 6C, D**). Furthermore, Hub DETh17RGs expression data were obtained from GSE142025 dataset and unsupervised hierarchical clustering was conducted using ward.D2 method. The results showed that most of the early DN samples were divided into cluster 1 (5/6,83%), while the vast majority of late DN samples were classified as cluster 2 (20/21,95%), which fully demonstrated that Hub DETh17RGs can well distinguish between early DN and advanced DN (**Figure 6E**). Details of all datasets used in this study are displayed in **Table 2**.

**Table 2:** Datasets implemented for analysis in this study.

Dataset	Platform	Case samples	Control samples
GSE160306 (23)	GPL20301	10	9
GSE94019 (24)	GPL11154	9	4
GSE102485 (25)	GPL18573	22	3
GSE60436 (26)	GPL6884	6	3
GSE30528 (27)	GPL571	9	13
GSE96804 (28)	GPL17586	41	20
GSE142025 (29)	GPL20301	21	6

### Verification of Relative Expression of Hub DETh17RGs by Quantitative Real-Time Polymerase Chain Reaction

**Figure 7:**
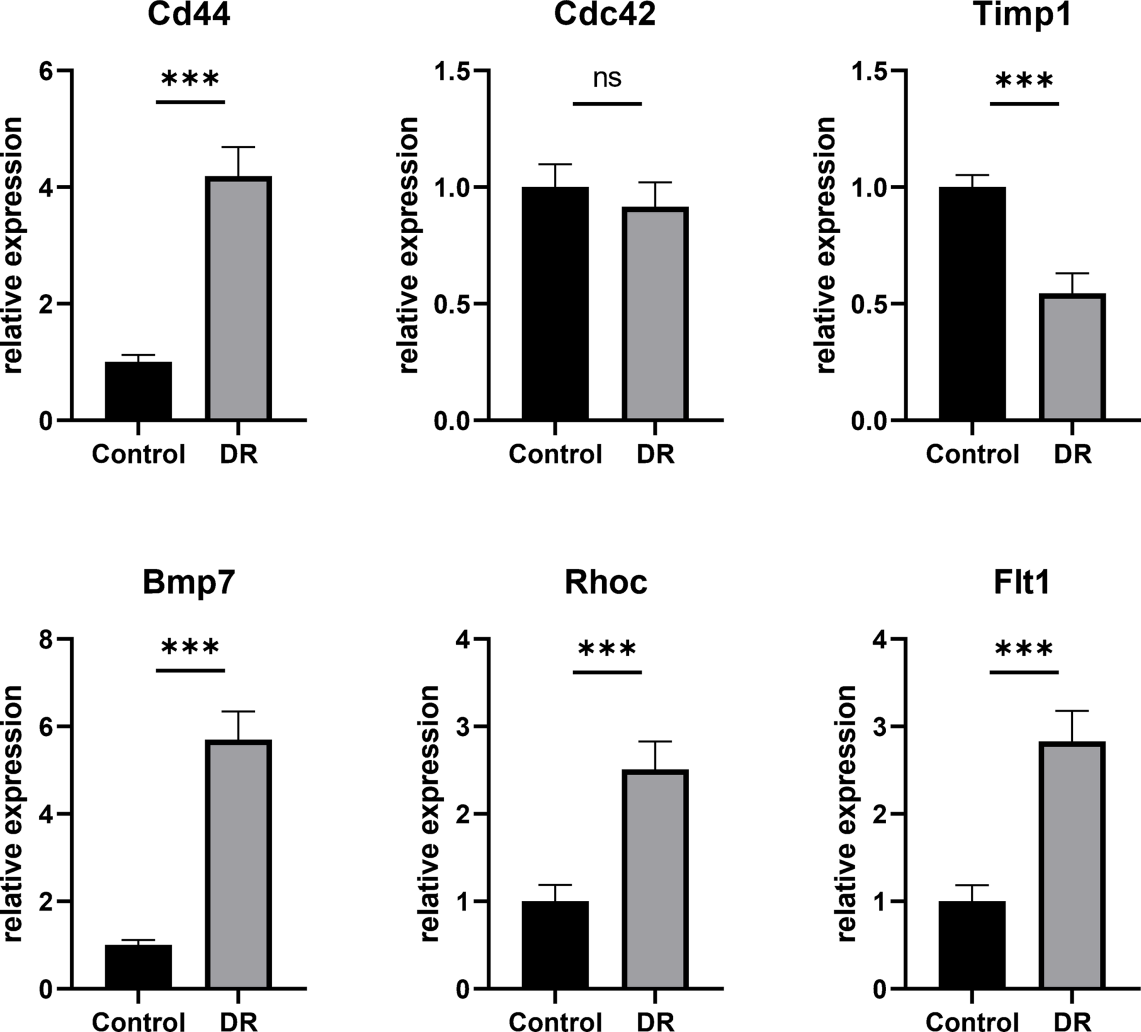
The Hub DETh17RGs mRNA levels in the retina of DR mouse models and controls. Compared with the control group, the transcription levels of Cd44, Bmp7, Rhoc and Flt1 in the DR mouse model group were significantly up-regulated, while the changes of Cdc42 were not significant, and the transcription level of Timp1 was significantly down-regulated. ***p < 0.001, NS *p* > 0.05.

In order to verify the results of comprehensive bioinformatic analysis, qPCR analysis was performed on the retinal samples of the mice in the study. The results illustrated that Cd44, Bmp7, Rhoc and Flt1 in DR group were up-regulated by 4.19, 5.70, 2.51 and 2.83 times, respectively, compared with the control group, which were consistent with the results of transcriptome analysis in this study with significant differences. There was no significant difference in Cdc42 expression between the two groups. Although there was significant difference in Timp1 between the two groups, it was down-regulated compared to the control group, which was different from the results in our transcriptome analysis (**Figure 7**). The above animal experiments results were generally consistent with the transcriptome analysis results in this study, which proves the reliability of the transcriptome data.

## Discussion

Recent studies have illustrated that differentiated and mature Th17 cells participate in the process of innate and acquired immunity by promoting the secretion of cytokines with strong pro-inflammatory effects, such as IL-17A, IL-17F, IL-21, IL-22, IL-26, etc., leading to inflammatory damage of tissues and organs, and play an important role in the formation and development of inflammatory diseases (30–32). The abnormality of Th17 cells is one of the momentous reasons for the progression of diabetes (33), however, the study of its mechanism in DR is still in its infancy. Some studies have confirmed that the level of serum IL-17 in patients with DR is increased (34, 35), the level of IL-17A in vitreous fluid of patients with PDR is increased, but there is no significant change in aqueous humor (36, 37). Animal experiments have revealed that the immune response mediated by Th17 cells plays a promoting role in the morphological and functional changes of retinal vessels in spontaneous diabetes mellitus (38). However, in the development of DR, especially DME, the molecular mechanism and genetic markers of Th17 in the retina, especially in macular retina, need to be studied. In our study, the score of Th17RG in DME was significantly higher than that in NPDR, which indirectly proved that the number of macular Th17 cells in DME was significantly higher than that in NPDR. Through the functional analysis and annotation of DETh17RGs, the results certified that they were mainly enriched in cell movement, migration, proliferation, response to stimulation, immune response, signal transduction and regulation; complement and coagulation cascade, leukocyte transendothelial migration, neutrophil extracellular trap formation, and some pivotal pathways. Through a series of algorithm calculations and verifications, CD44, CDC42, TIMP1, BMP7, RHOC and FLT1 were sieved as Hub DETh17RGs from 238 DETh17RGs. Verification of multiple independent datasets confirmed that they could identify different stages of DR disease. More interestingly, the hub gene features constructed by aggregating six genes by GSVA can distinguish not only early DN and late DN patients, but also DN patients and healthy people.

One of the most important functions of immune cells is the migration during development, inflammation and homeostasis (39). Th17 cells also follow this rule. They are highly efficient inflammatory cells that can trigger tissue inflammation and induce other inflammatory cells to infiltrate target organs (40, 41). Some studies have presented that Th17 cells infiltrate into the intraocular tissue from the circulatory system through the retinal vascular endothelium and participate in the inflammatory response in the retinal pigment epithelium (RPE) and uvea (42, 43). Other studies have confirmed that intercellular adhesion molecule-1 mediates the migration of Th17 cells in human retinal vascular endothelial cells (44). The co-staining of IL-17, RORγt and CD4 in the retina of diabetic retinopathy showed that Th17 cells infiltrated and damaged the retina during the formation of DR (45). The above results are consistent with the top five GO functional enrichment results in this study: the top five terms enriched by DETh17RGs in this study are all related to cell movement, migration and regulation. Th17 cells themselves belong to a subcategory of inflammatory T helper cells, so it is natural to demonstrate their involvement in the immune response and immune system processes in the GO enrichment analysis.

In addition, DETh17RGs is also enriched in many pathways related to DR and DME, such as hedgehog signaling pathway, complement and coagulation cascade, Wnt signaling pathway, p53 signaling pathway, Rap1 signaling pathway, NF-κB signaling pathway, leukocyte transendothelial migration, nod-like receptor signaling pathway, extracellular trap formation of neutrophils and so on. The complement and coagulation cascade reaction is a complex biological process, which is the two main proteolysis cascade reactions in blood (46, 47). Complement-coagulation interactions are essential for the appropriate innate response to retinal injury caused by diabetes, which can limit the development of hyperemia and inflammation while promoting healing (48, 49). Several studies have shown that the complement and coagulation cascade are related to DR, which is reflected in the accumulation of many coagulation factors and complement-related genes such as C1QB, C1QC, C3 and C9. The differential expression of C1QB and C1QC was also verified in DETh17RGs in our study (50, 51). Platelet endothelial cell adhesion molecule-1 (PECAM-1), which is highly expressed in the intercellular junction of retinal endothelial cells and the lumen-facing region of blood vessels, is an indispensable participant in the migration of leukocytes across the endothelium to the retinal tissue (52). However, its expression in DR retinal tissue is reduced, which can cause leukocyte stasis, lead to normal capillary blockage, distal normal capillary degeneration, hypoxia in the supply area, and stimulate neovascularization (53). At the same time, due to the increase of vascular capillary permeability caused by long-term hyperglycemia, immune cells such as neutrophils become easier to infiltrate into choroid and retina through capillaries, leading to the progression of retinopathy caused by this chronic inflammation (54). *In vivo* and *in vitro* studies have reported that high glucose can increase the release of Neutrophil extracellular traps (NET) (55). It has also been reported that the level of NET biomarkers is an independent risk factor for DR, and the presence of NET complex in DM patients with PDR was positively correlated with the severity of DR (55, 56). Multiple factors coordination mediate the ability of neutrophils to exert NET, which has recently been found to be the core participant in the pathogenesis of PDR induced by DM (57, 58). The above facts are highly consistent with the formation of extracellular traps of neutrophils analyzed in this study. A number of studies have confirmed that signaling pathways, such as Rap1, NF-κB and NOD, are involved in the pathogenesis of DR and DME and are crucial in disease formation (59–61); others have confirmed that in the formation of DR and DME, signaling pathways like hedgehog, PI3K and Wnt even have crosstalk mechanism (62). Our study reveals the possible signaling pathways related to Th17 cell infiltration and DME formation, which provides a basis for follow-up research.

In this study, six hub DETh17RGs were identified from the PPI network, which are CD44, CDC42, TIMP1, BMP7, RHOC and FLT1. Gene-encoded proteins participate in the occurrence and development of DR and have a significant impact on the disease process. CD44 is an extensively expressed cell surface adhesion molecule, which subserves pathological angiogenesis and disease evolution and the development of DR by regulating endothelial cell adhesion, invasion, proliferation, migration and intercellular communication (63). It was also verified in *in vitro* assays that CDC42 promotes cytoskeletal rearrangement, cell differentiation, and cell proliferation by controlling signaling transduction pathways, while also regulating cell migration and cell adhesion (64–66). Animal studies have approved the importance of CDC42 in filamentous pseudopodia formation and endothelial cell migration (67); it has been confirmed that CDC42 is also involved in diabetes-related complications, such as insulin resistance, DN, diabetic cardiomyopathy, and further research in DR is underway (68). TIMP-1 is a strong inhibitor of most matrix metalloproteinases (MMP), and the balance between them may be critical to tissue homeostasis in DR (69). Studies have reported elevated levels of TIMP-1 in vitreous fluid in the PDR, and some studies have shown that the balance between TIMP-1 and MMP may be disrupted early in the onset of DR (69–71). In late NPDR/PDR, the concentration of TIMP-1 in aqueous humor increased, but there was no significant change in serum, indicating that its intraocular regulation was independent of systemic regulation, and that TIMP-1 was involved in the progression of DR (71). BMP-7, a member of BMPs, interacts with TGF- β and participates in the process of tissue degeneration and fibrosis. Many studies have demonstrated the antagonistic and reverse fibrosis effects of BMP-7 in fibrosis, so it is reasonable to suggest that BMP-7 may have the same preventive effect on ocular fibrotic diseases such as DR (72). It has been reported that BMPs acts as a regulator of the EMT process in DR and plays a role in angiogenesis and inflammation (73). The overexpression of RHOC is associated with cell proliferation that enhances cell motility, makes it invasive, and promotes the production of angiogenic factors such as VEGF (74, 75). By controlling the proliferation, migration and vascular permeability of endothelial cells, RHOC plays a role in regulating angiogenesis in DR and related macular disease, as well as in maintaining vascular homeostasis (75, 76). FLT1, the first member of the vascular endothelial growth factor receptor (VEGFR) family, also known as VEGFR1, plays multiple diverse roles in vascular development, angiogenesis, cell survival and inflammation (77, 78). Studies have confirmed that VEGFR1 is involved in the pathogenesis of DR and DME, and relevant drugs that target it have been developed to treat DR and DME (78, 79). In this study, the results of two genes, CDC42 and TIMP1, are inconsistent between RNA-seq and qPCR. We consider that there may be the following reasons: first, the quantification of qPCR is measured in the local region of the gene, while the quantification of RNA-seq is measured in the full-length region of the gene. Therefore, the difference in the quantity of gene expression between qPCR and RNA-seq will lead to conflict in estimating the change of gene expression level, but it does not mean that the result of one of these methods is wrong. Second, similarly, if there is a difference in gene expression in RNA-seq, but no difference is detected in qPCR, the designed qPCR primers may amplify a region of exons with no significant change in expression. Third, RNA-seq and qPCR themselves are two different experimental platforms, and it is reasonable that some of the results can not be completely one-to-one correspondence because of the different technical principles. Studies have confirmed that, in strict accordance with the analytical workflow, 15-20% of genes are considered to be “inconsistent” when comparing the results obtained with RNA-seq with those obtained with qPCR (“inconsistency” is defined as differential expression between two methods in the opposite direction, or one method shows differential expression while the other does not) (80).

In terms of diabetic microvascular complications, DR and DN have similar pathogenesis, retinal vessels and renal vessels have similar physiological structures, both of which can reflect microcirculation injury (81). Various metabolic disorders in patients with diabetes can activate a variety of pathophysiological processes in the body and promote the occurrence of microvascular complications. Under the condition of high glucose, the polyol pathway begins to participate in glucose metabolism and aldose reductase reduces intracellular glucose to insoluble sorbitol, thus changing the permeability of the cell membrane and causing a large amount of water to enter kidney and retinal endothelial cells rapidly, resulting in edema and cell damage. Furthermore, advanced glycation end products (AGE) interact with their receptors to increase intracellular oxidative stress, activate NF-κB, and eventually cause inflammation, resulting in renal and retinal vascular dysfunction (82). In addition, increasing intracellular glucose concentration leads to increased diacylglycerol (DAG), the key helper of protein kinase C (PKC), which activates the PKC pathway and increases the expression of vasoconstrictor endothelin-1 to reduce blood flow, resulting in thickening of the renal basement membrane, increased extracellular matrix, and retinal neovascularization (82, 83). Although it is impossible to determine the order of onset of DR and DN in the clinic, the phenomenon that both exist at the same time is more common in clinic (84). Clinically, DR and DN are mutually independent risk factors, which influence the development and change of the disease (19, 85, 86). Common risk factors for the progression of DR and DN include blood glucose, blood pressure, blood lipids, course of diabetes, obesity, smoking and so on (87). At present, a number of studies have shown that DR has diagnostic value for DN: meta-studies have shown that DR has a sensitivity of 0.65 and a specificity of 0.75 in distinguishing DN from non-diabetic renal disease (NDRD) in patients with type 2 diabetes, and they emphasized that PDR has a high specificity in the diagnosis of DN (88). In addition, the latest meta-analysis revealed that the sensitivity and specificity of DR in diagnosing DN were 0.67 and 0.78, and the specificity of PDR in predicting DN was 0.99, suggesting that DR is a good index to predict DN (89). A study on the predictive efficacy of the severity of retinopathy on end-stage renal disease (ESRD) in patients with DN discovered that 56% of DN patients with PDR progressed to ESRD after a median follow-up of 15 months, suggesting that the severity of DR was an independent risk factor for ESRD (90). A study in Taiwan, China, followed 4,050 patients with chronic kidney disease (CKD) for at least one year and found that DR was a risk factor for the progression of CKD, while patients with PDR had a significantly higher risk of progression of CKD than patients with NPDR at baseline, with an OR of 2.18 (91). Another study showed that the severity of DR in patients with type 2 diabetes was positively correlated with the progression of CKD, and the risk of CKD progression in patients with PDR was 16.6 times higher than that in patients without DR (92). Macular edema refers to the abnormal increase of macular fluid and the infiltration of extracellular fluid into the retinal capsule, when there is thickening in the nonvascular area of the macular fovea or rigid exudation and thickening within 500 μm from the fovea. It is called clinically significant macular edema (93). Studies have found that patients with clinically significant macular edema are more likely to develop nephropathy (94). In addition, some researchers divided macular edema into diffuse, cystic, serous, vitreous traction and mixed cystoid and serous types according to their morphology. The results demonstrated that serous macular edema had the highest probability of albuminuria, and severe renal failure was related to macular edema (94). It can be seen that DR patients with macular edema, especially with the clinical significance of serous macular edema, have a higher probability of nephropathy. In recent years, there have been few studies on the relationship between macular edema and nephropathy, and some studies have indicated that there is no correlation between macular edema and eGFR (95). This may be due to the occurrence of diabetic macular edema in the late stage of DR, and the consistency of DN and DR in the course of disease. Therefore, early observation of macular edema and eGFR may not be able to find a strong relationship. However, when patients with diabetes have both DR and macular edema, they are more likely to be diagnosed with DN. Although the role of Hub DETh17RGs in DR has been generally revealed, there is still a lack of research on their respective mechanisms at the DME level. Our study illustrated that their expression patterns between NPDR and DME are basically the same as those of normal and PDR in independent datasets. This phenomenon demonstrated that Hub DETh17RGs in DR is not only related to the occurrence of DME in the macula, but also to the progression of the disease, which is also confirmed by the brief overview of the relationship between each Hub DETh17RG and DR. The most significant finding is that the expression pattern of Hub DETh17RGs at different stages of DN is similar to that of DR in this study: It can distinguish not only early DN and late DN patients, but also DN patients and healthy people. Therefore, Hub DETh17RGs excavates the clinically observed macro-relationship between DR, DME and DN to the micro-genetic level, and also reflects its diagnostic value in two different organs of diabetic complications.

Our research still has some limitations. First of all, in the course of long-term chronic disease of diabetes, the gene expression patterns of some immune cell types may be adjusted due to the changes of complex pathological environment around different tissues. In this study, immune cells located in glomerulus and macula showed different expression profiles under the regulation of tissue microenvironment. Due to the influence of this factor, we can only build tissue infiltration-specific model separately according to tissue types, but cannot combine the immune cells of two different tissues collected to build a unified model. Therefore, data cannot be combined as a whole to complete data comparison between multiple groups of tissues. Secondly, because some of the immune cells in the study belong to certain cell subsets, they may have similar transcriptome characteristics or share some common characteristic genes, and these types of cells may potentially interfere with the calculation of proportional scores. Thirdly, because the type of the original dataset belongs to the case-control study, it is impossible to clarify the causal relationship between the expression of DETh17RGs and the abundance of Th17 cells. Fourth, a prospective cohort study is needed in diabetic patients without complications such as DR, DME and DN to verify the diagnostic and prognostic value of Hub DETh17RGs. Finally, no *in vitro* experimental studies have been carried out to confirm our findings, and *in vivo* experiments need to be further explored. In the future research, a large number of samples are needed to study the pathogenesis of cells and animals at the molecular biological level in order to elucidate the crosstalk mechanism of Hub DETh17RGs in different organs and tissues of diabetes.

## Conclusion

Through comprehensive bioinformatic analysis, this study identified the Th17 cell-related genes closely associated with the progression of DME, and also analyzed the possible molecular mechanisms linking Th17 cells with the progression of DME and DR. The Hub DETh17RGs obtained by implementing a variety of reliable algorithms plays a critical role in the occurrence and progression of DME, and they may also play a pivotal role in the disease progression of DN. These results expand our understanding of the mechanisms underlying the effects of Th17 cells on diabetic complications such as DR, DME and DN, and demonstrate their potential as new therapeutic targets for DR and DME.

## Data Availability Statement

The datasets presented in this study can be found in online repositories. The names of the repository/repositories and accession number(s) can be found in the article/supplementary material.

## Ethics Statement

The animal study was reviewed and approved by Medical Research Ethics Committee of First Affiliated Hospital of Nanchang University.

## Author Contributions

JH performed the data analysis and drafted the manuscript. JH and QZ contributed to the revising of this manuscript. JH conceived and designed the experiments and revised the manuscript. All authors have seen and approved the final manuscript. All authors contributed to the article and approved the submitted version

## Funding

This study was supported by the Key research and development project in Jiangxi Province, No. 20192BBGL70033; Key research and development project in Jiangxi Province, No. 20203BBG73058 and The Central Government Guides Local Science and Technology Development Foundation, No. 20211ZDG02003.

## Conflict of Interest

The authors declare that the research was conducted in the absence of any commercial or financial relationships that could be construed as a potential conflict of interest.

## Publisher’s Note

All claims expressed in this article are solely those of the authors and do not necessarily represent those of their affiliated organizations, or those of the publisher, the editors and the reviewers. Any product that may be evaluated in this article, or claim that may be made by its manufacturer, is not guaranteed or endorsed by the publisher.
